# Diammonium bis­[(2-amino­acetato-κ^2^ *N*,*O*)(2,2′-bipyridine-κ^2^ *N*,*N*′)(*N*,*N*-dimethyl­formamide-κ*O*)copper(II)] hexa­cosa­oxidoocta­molybdate(VI)

**DOI:** 10.1107/S1600536808000020

**Published:** 2008-01-09

**Authors:** Haiyan Liu, Yunjie Zhang, Decheng Yu

**Affiliations:** a College of Chemistry and Pharmacy, Jiamusi University, Jiamusi 154007, People’s Republic of China

## Abstract

The title compound, (NH_4_)_2_[Cu(C_2_H_4_NO_2_)(C_10_H_8_N_2_)(C_3_H_7_NO)]_2_[Mo_8_O_26_], contains a centrosymmetric β-type octa­molybdate anion, two copper(II) complex cations and two ammonium ions. The Cu^II^ atom is coordinated in a square-pyramidal geometry by a 2,2′-bipyridine and a 2-amino­acetate ligands in the basal plane and by an O atom of *N*,*N*-dimethyl­formamide in the apical position. The anions and cations are linked by N—H⋯O hydrogen bonds into a three-dimensional network.

## Related literature

For related literature, see: Allis *et al.* (2004 ▶); Brown & Altermatt (1985 ▶).
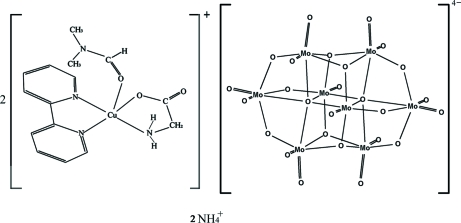


## Experimental

### 

#### Crystal data

(NH_4_)_2_[Cu(C_2_H_4_NO_2_)(C_10_H_8_N_2_)(C_3_H_7_NO)]_2_[Mo_8_O_26_]
*M* *_r_* = 1953.38
Triclinic, 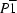

*a* = 10.222 (2) Å
*b* = 10.849 (2) Å
*c* = 13.020 (3) Å
α = 81.82 (3)°
β = 69.91 (2)°
γ = 81.61 (3)°
*V* = 1334.9 (5) Å^3^
*Z* = 1
Mo *K*α radiation
μ = 2.69 mm^−1^
*T* = 291 (2) K
0.28 × 0.20 × 0.14 mm

#### Data collection

Bruker SMART APEXII diffractometer
Absorption correction: multi-scan (*SADABS*; Sheldrick, 1996 ▶) *T*_min_ = 0.530, *T*_max_ = 0.690
10864 measured reflections
4889 independent reflections
3591 reflections with *I* > 2σ(*I*)
*R*_int_ = 0.070

#### Refinement

*R*[*F* ^2^ > 2σ(*F* ^2^)] = 0.046
*wR*(*F* ^2^) = 0.110
*S* = 0.99
4889 reflections
370 parameters
H-atom parameters constrained
Δρ_max_ = 0.86 e Å^−3^
Δρ_min_ = −0.83 e Å^−3^

### 

Data collection: *APEX2* (Bruker, 2004 ▶); cell refinement: *SAINT* (Bruker, 2004 ▶); data reduction: *SAINT*; program(s) used to solve structure: *SHELXTL* (Bruker, 1997 ▶); program(s) used to refine structure: *SHELXTL*; molecular graphics: *DIAMOND* (Brandenburg, 1999 ▶); software used to prepare material for publication: *SHELXTL* (Bruker, 1997 ▶.

## Supplementary Material

Crystal structure: contains datablocks I, global. DOI: 10.1107/S1600536808000020/hy2114sup1.cif

Structure factors: contains datablocks I. DOI: 10.1107/S1600536808000020/hy2114Isup2.hkl

Additional supplementary materials:  crystallographic information; 3D view; checkCIF report

## Figures and Tables

**Table 1:** Selected bond lengths (Å)

Cu—O14	1.925 (4)
Cu—O16	2.604 (4)
Cu—N1	1.985 (6)
Cu—N2	1.989 (6)
Cu—N3	1.990 (6)
Mo1—O1	1.705 (5)
Mo1—O6	1.720 (4)
Mo1—O11	1.896 (4)
Mo1—O10	1.995 (5)
Mo1—O12	2.306 (4)
Mo1—O7^i^	2.356 (5)
Mo2—O8	1.699 (4)
Mo2—O2	1.722 (5)
Mo2—O9	1.897 (4)
Mo2—O7^i^	1.998 (5)
Mo2—O10	2.340 (4)
Mo2—O12^i^	2.346 (4)
Mo3—O4	1.696 (5)
Mo3—O5	1.756 (5)
Mo3—O10	1.960 (4)
Mo3—O7	1.967 (4)
Mo3—O12	2.143 (4)
Mo3—O12^i^	2.380 (4)
Mo4—O13	1.693 (4)
Mo4—O3	1.716 (5)
Mo4—O9^i^	1.921 (4)
Mo4—O11	1.945 (4)
Mo4—O5^i^	2.257 (5)
Mo4—O12	2.491 (4)

Symmetry code: (i) 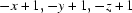.

**Table 2:** Hydrogen-bond geometry (Å, °)

*D*—H⋯*A*	*D*—H	H⋯*A*	*D*⋯*A*	*D*—H⋯*A*
N1—H1*A*⋯O3^ii^	0.90	2.32	3.093 (7)	144
N1—H1*B*⋯O6	0.90	2.01	2.863 (6)	158
N5—H*N*1⋯O1	0.90	2.10	2.888 (7)	146
N5—H*N*2⋯O4	0.95	2.13	3.028 (8)	158
N5—H*N*3⋯O15^iii^	0.95	1.94	2.761 (8)	143
N5—H*N*4⋯O14^iii^	0.98	2.26	3.132 (7)	148

Symmetry codes: (ii) 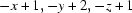; (iii) 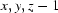.

**Fig. 1.**
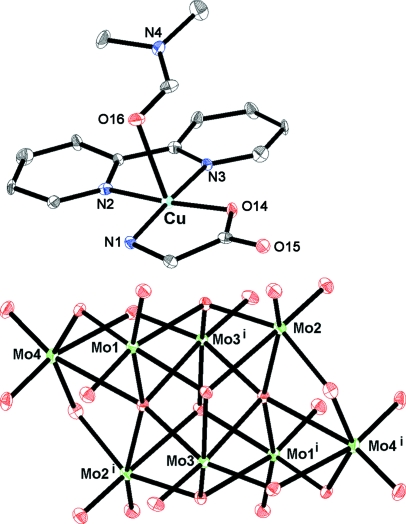
The structures of the anion and cation of the title compound (the ammonium ion has been omitted). Displacement ellipsoides are drawn at the 30% probability level. [Symmetry code: (i) 1 - x, 1 - y, 1 - z.]

**Fig. 2.**
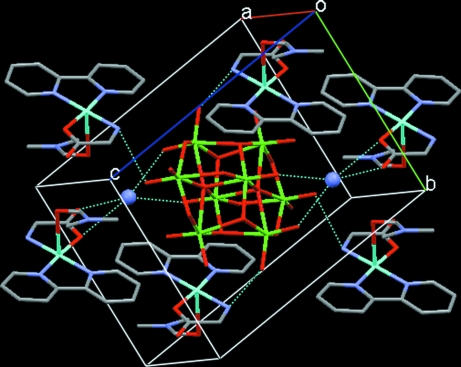
Crystal packing of the title compound. The ammonium ions are drawn as ball. Dotted lines denote hydrogen bonds.

## supplementary crystallographic information

### Comment

Polymolybdates (POMs) modified by transition metal complexes have been extensively explored (Allis *et al.*, 2004). To further explore new POMs belonging to this family, the title compound is reported here.

The title compound consists of a [Mo_8_O_26_]^4-^ anion, two [Cu(bpy)(gly)(dmf)]^+^ cations and two ammonium ions (bpy = 2,2'-bipyridine, gly = 2-aminoacetate, dmf = *N*,*N*-dimethylfomamide) (Fig. 1). The [Mo_8_O_26_]^4-^ anion in the β-type lies on an inversion center, made up of eight edge-sharing MoO_6_ octahedra. The Cu^II^ atom in the [Cu(bpy)(gly)(dmf)]^+^ cation displays a distorted square-pyramidal geometry with two N atoms from the 2,2'-bipyridine ligand and an O atom and an N atom from the 2-aminoacetate ligand in the basal plane and an O atom of *N*,*N*-dimethylfomamide in the apical position. The Mo—O, Cu—N and Cu—O distances are given in Table 1. A calculation of bond valence sum (Brown & Altermatt, 1985) indicates the oxidation states of 5.71–5.84 for the Mo atoms and 2.17 for the Cu atom, in agreement with the expected values. These anions and cations connect to each other by N—H···O hydrogen bonds (Table 2), forming a three-dimensional supramolecular network, as shown in Fig. 2.

### Experimental

(NH_4_)_6_(Mo_7_O_24_).4H_2_O (1.50 g, 1.2 mmol), Cu(CH_3_COO)_2_.2H_2_O (0.20 g, 1.0 mmol), 2-aminoacetic acid (0.075 g, 1.0 mmol) and bpy (0.16 g, 1.0 mmol) were dissolved in 0.5 *M* HCl solution (15 ml) with stirring. Then the suspension was added to a dmf solution (10 ml), which was refluxed at 333 K for 3 h. After cooling to room temperature, the mixture was filtrated and transferred into a 50 ml beaker. Blue block crystals of the title compound suitable for X-ray diffraction were obtained after several days.

### Refinement

H atoms of ammonium were located in difference Fourier maps and fixed in their as-found positions (N—H = 0.90–0.98 Å) with *U*_iso_(H) = 1.2*U*_eq_(N). The other H atoms were positioned geometrically and refined as riding atoms, with C—H = 0.93Å (CH), 0.97Å (CH_2_), N—H = 0.90Å and *U*_iso_(H) = 1.2*U*_eq_(C,*N*), and with C—H = 0.96Å (CH_3_) and *U*_iso_(H) = 1.5*U*_eq_(C).

### Crystal data

**Table:**

(NH_4_)_2_[Cu(C_2_H_4_NO_2_)(C_10_H_8_N_2_)(C_3_H_7_NO)]_2_[Mo_8_O_26_]	*Z* = 1
*M**_r_* = 1953.38	*F*_000_ = 946
Triclinic, *P*1	*D*_x_ = 2.430 Mg m^−^^3^
Hall symbol: -P 1	Mo *K*α radiation λ = 0.71073 Å
*a* = 10.222 (2) Å	Cell parameters from 3544 reflections
*b* = 10.849 (2) Å	θ = 1.6–26.3º
*c* = 13.020 (3) Å	µ = 2.70 mm^−^^1^
α = 81.82 (3)º	*T* = 291 (2) K
β = 69.91 (2)º	Block, blue
γ = 81.61 (3)º	0.28 × 0.20 × 0.14 mm
*V* = 1334.9 (5) Å^3^	

### Data collection

**Table:**

Bruker SMART APEXII diffractometer	4889 independent reflections
Radiation source: fine-focus sealed tube	3591 reflections with *I* > 2σ(*I*)
Monochromator: graphite	*R*_int_ = 0.071
*T* = 291(2) K	θ_max_ = 25.6º
φ and ω scans	θ_min_ = 3.0º
Absorption correction: multi-scan(SADABS; Sheldrick, 1996)	*h* = −12→12
*T*_min_ = 0.530, *T*_max_ = 0.690	*k* = −13→13
10864 measured reflections	*l* = −15→14

### Refinement

**Table:**

Refinement on *F*^2^	Secondary atom site location: difference Fourier map
Least-squares matrix: full	Hydrogen site location: inferred from neighbouring sites
*R*[*F*^2^ > 2σ(*F*^2^)] = 0.046	H-atom parameters constrained
*wR*(*F*^2^) = 0.110	*w* = 1/[σ^2^(*F*_o_^2^) + (0.0226*P*)^2^] where *P* = (*F*_o_^2^ + 2*F*_c_^2^)/3
*S* = 0.99	(Δ/σ)_max_ < 0.001
4889 reflections	Δρ_max_ = 0.86 e Å^−^^3^
370 parameters	Δρ_min_ = −0.83 e Å^−^^3^
Primary atom site location: structure-invariant direct methods	Extinction correction: none

### Special details

**Table:**

Geometry. All e.s.d.'s (except the e.s.d. in the dihedral angle between two l.s. planes) are estimated using the full covariance matrix. The cell e.s.d.'s are taken into account individually in the estimation of e.s.d.'s in distances, angles and torsion angles; correlations between e.s.d.'s in cell parameters are only used when they are defined by crystal symmetry. An approximate (isotropic) treatment of cell e.s.d.'s is used for estimating e.s.d.'s involving l.s. planes.

### Fractional atomic coordinates and isotropic or equivalent isotropic displacement parameters (Å^2^)

**Table:**

	*x*	*y*	*z*	*U*_iso_*/*U*_eq_	
Cu	0.56754 (7)	0.78752 (8)	0.79555 (7)	0.0191 (2)	
Mo1	0.39065 (5)	0.75790 (5)	0.50257 (5)	0.01630 (15)	
Mo2	0.36042 (5)	0.54294 (5)	0.72689 (5)	0.01687 (15)	
Mo3	0.34518 (5)	0.48004 (5)	0.47185 (5)	0.01409 (15)	
Mo4	0.69102 (5)	0.74002 (5)	0.30214 (5)	0.01864 (16)	
O1	0.3028 (4)	0.8061 (4)	0.4114 (4)	0.0242 (11)	
O2	0.4582 (4)	0.4794 (4)	0.8097 (4)	0.0244 (11)	
O3	0.5990 (4)	0.7957 (4)	0.2141 (4)	0.0258 (11)	
O4	0.2585 (4)	0.5422 (4)	0.3833 (4)	0.0227 (11)	
O5	0.2406 (4)	0.3680 (4)	0.5603 (4)	0.0211 (11)	
O6	0.3191 (4)	0.8577 (4)	0.6038 (4)	0.0235 (11)	
O7	0.4909 (4)	0.3616 (4)	0.3840 (4)	0.0172 (10)	
O8	0.2517 (5)	0.6614 (4)	0.7940 (4)	0.0256 (11)	
O9	0.2357 (4)	0.4195 (4)	0.7536 (4)	0.0223 (11)	
O10	0.2874 (4)	0.6092 (4)	0.5743 (4)	0.0167 (10)	
O11	0.5653 (4)	0.8223 (4)	0.4285 (4)	0.0192 (10)	
O12	0.5191 (4)	0.5901 (4)	0.4106 (3)	0.0150 (9)	
O13	0.8335 (4)	0.8205 (5)	0.2580 (4)	0.0282 (12)	
O14	0.4272 (4)	0.7617 (4)	0.9377 (4)	0.0223 (11)	
O15	0.2086 (4)	0.8280 (4)	1.0374 (4)	0.0247 (11)	
O16	0.6474 (5)	0.9741 (5)	0.8567 (5)	0.0330 (13)	
N1	0.4294 (5)	0.9213 (5)	0.7618 (4)	0.0186 (12)	
H1A	0.4624	0.9962	0.7515	0.022*	
H1B	0.4162	0.9084	0.6994	0.022*	
N2	0.7255 (5)	0.8036 (5)	0.6548 (5)	0.0195 (13)	
N3	0.7002 (5)	0.6481 (5)	0.8305 (5)	0.0176 (12)	
N4	0.8488 (6)	0.8849 (6)	0.8898 (5)	0.0257 (14)	
N5	0.3354 (5)	0.7456 (6)	0.1944 (5)	0.0272 (14)	
HN1	0.3438	0.7903	0.2442	0.033*	
HN2	0.3330	0.6704	0.2418	0.033*	
HN3	0.2584	0.7693	0.1687	0.033*	
HN4	0.3996	0.7429	0.1189	0.033*	
C1	0.6771 (7)	0.5713 (7)	0.9239 (6)	0.0281 (18)	
H1	0.5909	0.5821	0.9791	0.034*	
C2	0.7758 (6)	0.4768 (6)	0.9418 (5)	0.0214 (15)	
H2	0.7570	0.4254	1.0076	0.026*	
C3	0.9044 (7)	0.4610 (7)	0.8582 (6)	0.0305 (17)	
H3	0.9719	0.3967	0.8667	0.037*	
C4	0.9304 (7)	0.5408 (7)	0.7636 (6)	0.0269 (17)	
H4	1.0174	0.5328	0.7089	0.032*	
C5	0.9663 (7)	0.7332 (7)	0.5632 (6)	0.0243 (16)	
H5	1.0468	0.6802	0.5620	0.029*	
C6	0.9666 (7)	0.8216 (8)	0.4778 (7)	0.037 (2)	
H6	1.0483	0.8279	0.4179	0.044*	
C7	0.8487 (7)	0.9025 (6)	0.4776 (6)	0.0242 (15)	
H7	0.8492	0.9621	0.4186	0.029*	
C8	0.7291 (6)	0.8904 (6)	0.5697 (6)	0.0202 (15)	
H8	0.6489	0.9446	0.5724	0.024*	
C9	0.8275 (6)	0.6337 (6)	0.7489 (6)	0.0200 (15)	
C10	0.8423 (6)	0.7234 (6)	0.6532 (5)	0.0177 (14)	
C11	0.2938 (6)	0.9214 (7)	0.8538 (6)	0.0216 (15)	
H11A	0.2230	0.8961	0.8293	0.026*	
H11B	0.2635	1.0053	0.8761	0.026*	
C12	0.3090 (6)	0.8328 (6)	0.9503 (6)	0.0213 (16)	
C13	0.7109 (7)	0.9109 (7)	0.9152 (7)	0.0316 (19)	
H14	0.6564	0.8782	0.9842	0.038*	
C14	0.9409 (7)	0.9421 (8)	0.7868 (7)	0.039 (2)	
H14A	1.0368	0.9144	0.7803	0.058*	
H14B	0.9257	1.0316	0.7863	0.058*	
H14C	0.9208	0.9179	0.7261	0.058*	
C15	0.9148 (8)	0.8121 (9)	0.9643 (7)	0.043 (2)	
H15A	1.0146	0.8049	0.9296	0.065*	
H15B	0.8833	0.7302	0.9825	0.065*	
H15C	0.8902	0.8530	1.0301	0.065*	

### Atomic displacement parameters (Å^2^)

**Table:**

	*U*^11^	*U*^22^	*U*^33^	*U*^12^	*U*^13^	*U*^23^
Cu	0.0149 (4)	0.0195 (5)	0.0211 (5)	0.0014 (3)	−0.0060 (3)	0.0001 (4)
Mo1	0.0182 (3)	0.0128 (3)	0.0184 (3)	0.0010 (2)	−0.0078 (2)	−0.0011 (2)
Mo2	0.0177 (3)	0.0176 (3)	0.0147 (3)	0.0002 (2)	−0.0052 (2)	−0.0019 (2)
Mo3	0.0135 (3)	0.0140 (3)	0.0155 (3)	−0.0008 (2)	−0.0062 (2)	−0.0008 (2)
Mo4	0.0185 (3)	0.0172 (3)	0.0199 (3)	−0.0037 (2)	−0.0073 (2)	0.0036 (3)
O1	0.027 (2)	0.023 (3)	0.024 (3)	0.004 (2)	−0.012 (2)	−0.002 (2)
O2	0.027 (2)	0.026 (3)	0.018 (3)	0.001 (2)	−0.006 (2)	−0.003 (2)
O3	0.027 (3)	0.020 (3)	0.029 (3)	−0.002 (2)	−0.012 (2)	0.009 (2)
O4	0.019 (2)	0.029 (3)	0.023 (3)	0.004 (2)	−0.011 (2)	−0.007 (2)
O5	0.017 (2)	0.021 (3)	0.027 (3)	−0.0034 (19)	−0.009 (2)	−0.001 (2)
O6	0.030 (3)	0.019 (3)	0.024 (3)	0.002 (2)	−0.012 (2)	−0.005 (2)
O7	0.021 (2)	0.013 (2)	0.020 (2)	0.0024 (18)	−0.0121 (19)	0.001 (2)
O8	0.031 (3)	0.021 (3)	0.025 (3)	0.003 (2)	−0.012 (2)	−0.005 (2)
O9	0.017 (2)	0.025 (3)	0.023 (3)	0.0033 (19)	−0.007 (2)	−0.002 (2)
O10	0.017 (2)	0.018 (2)	0.016 (2)	0.0007 (18)	−0.0081 (19)	−0.001 (2)
O11	0.022 (2)	0.016 (2)	0.021 (3)	−0.0067 (18)	−0.008 (2)	0.002 (2)
O12	0.014 (2)	0.015 (2)	0.016 (2)	−0.0009 (17)	−0.0053 (19)	0.0000 (19)
O13	0.026 (3)	0.026 (3)	0.034 (3)	−0.012 (2)	−0.012 (2)	0.008 (2)
O14	0.015 (2)	0.029 (3)	0.016 (3)	−0.0027 (19)	−0.0006 (19)	0.006 (2)
O15	0.021 (2)	0.028 (3)	0.023 (3)	−0.002 (2)	−0.006 (2)	−0.002 (2)
O16	0.036 (3)	0.031 (3)	0.042 (3)	−0.012 (2)	−0.023 (3)	0.000 (3)
N1	0.022 (3)	0.019 (3)	0.015 (3)	−0.002 (2)	−0.006 (2)	−0.002 (3)
N2	0.019 (3)	0.015 (3)	0.027 (3)	0.001 (2)	−0.010 (3)	−0.006 (3)
N3	0.020 (3)	0.013 (3)	0.021 (3)	−0.001 (2)	−0.011 (2)	0.003 (3)
N4	0.027 (3)	0.026 (3)	0.024 (3)	−0.010 (3)	−0.007 (3)	0.001 (3)
N5	0.029 (3)	0.034 (4)	0.019 (3)	0.002 (3)	−0.012 (3)	0.004 (3)
C1	0.022 (3)	0.026 (4)	0.034 (4)	0.004 (3)	−0.006 (3)	−0.006 (4)
C2	0.026 (3)	0.020 (4)	0.014 (3)	−0.005 (3)	−0.005 (3)	0.010 (3)
C3	0.029 (4)	0.032 (5)	0.027 (4)	0.006 (3)	−0.013 (3)	0.005 (4)
C4	0.018 (3)	0.026 (4)	0.029 (4)	−0.001 (3)	−0.001 (3)	0.006 (3)
C5	0.020 (3)	0.027 (4)	0.020 (4)	0.003 (3)	−0.005 (3)	0.006 (3)
C6	0.020 (4)	0.042 (5)	0.042 (5)	−0.001 (3)	−0.006 (4)	0.000 (4)
C7	0.027 (4)	0.020 (4)	0.023 (4)	−0.009 (3)	−0.008 (3)	0.012 (3)
C8	0.018 (3)	0.015 (3)	0.027 (4)	0.000 (3)	−0.009 (3)	0.001 (3)
C9	0.020 (3)	0.018 (4)	0.023 (4)	0.000 (3)	−0.008 (3)	−0.005 (3)
C10	0.020 (3)	0.018 (4)	0.015 (3)	−0.003 (3)	−0.007 (3)	0.001 (3)
C11	0.021 (3)	0.022 (4)	0.022 (4)	0.003 (3)	−0.009 (3)	−0.005 (3)
C12	0.022 (3)	0.022 (4)	0.024 (4)	0.000 (3)	−0.011 (3)	−0.008 (3)
C13	0.029 (4)	0.031 (5)	0.040 (5)	−0.008 (3)	−0.013 (4)	−0.011 (4)
C14	0.033 (4)	0.046 (5)	0.031 (4)	−0.021 (4)	0.002 (4)	0.004 (4)
C15	0.031 (4)	0.054 (6)	0.038 (5)	0.010 (4)	−0.009 (4)	−0.001 (5)

### Geometric parameters (Å, °)

**Table:**

Cu—O14	1.925 (4)	N2—C10	1.367 (8)
Cu—O16	2.604 (4)	N3—C1	1.343 (10)
Cu—N1	1.985 (6)	N3—C9	1.373 (8)
Cu—N2	1.989 (6)	N4—C13	1.329 (8)
Cu—N3	1.990 (6)	N4—C15	1.447 (10)
Mo1—O1	1.705 (5)	N4—C14	1.462 (9)
Mo1—O6	1.720 (4)	N5—HN1	0.8954
Mo1—O11	1.896 (4)	N5—HN2	0.9477
Mo1—O10	1.995 (5)	N5—HN3	0.9466
Mo1—O12	2.306 (4)	N5—HN4	0.9785
Mo1—O7^i^	2.356 (5)	C1—C2	1.384 (10)
Mo1—Mo3	3.2161 (10)	C1—H1	0.9300
Mo2—O8	1.699 (4)	C2—C3	1.395 (9)
Mo2—O2	1.722 (5)	C2—H2	0.9300
Mo2—O9	1.897 (4)	C3—C4	1.370 (11)
Mo2—O7^i^	1.998 (5)	C3—H3	0.9300
Mo2—O10	2.340 (4)	C4—C9	1.390 (9)
Mo2—O12^i^	2.346 (4)	C4—H4	0.9300
Mo3—O4	1.696 (5)	C5—C6	1.361 (12)
Mo3—O5	1.756 (5)	C5—C10	1.405 (9)
Mo3—O10	1.960 (4)	C5—H5	0.9300
Mo3—O7	1.967 (4)	C6—C7	1.383 (10)
Mo3—O12	2.143 (4)	C6—H6	0.9300
Mo3—O12^i^	2.380 (4)	C7—C8	1.395 (9)
Mo4—O13	1.693 (4)	C7—H7	0.9300
Mo4—O3	1.716 (5)	C8—H8	0.9300
Mo4—O9^i^	1.921 (4)	C9—C10	1.446 (10)
Mo4—O11	1.945 (4)	C11—C12	1.508 (10)
Mo4—O5^i^	2.257 (5)	C11—H11A	0.9700
Mo4—O12	2.491 (4)	C11—H11B	0.9700
O14—C12	1.307 (7)	C13—H14	0.9300
O15—C12	1.242 (8)	C14—H14A	0.9600
O16—C13	1.239 (10)	C14—H14B	0.9600
N1—C11	1.488 (8)	C14—H14C	0.9600
N1—H1A	0.9000	C15—H15A	0.9600
N1—H1B	0.9000	C15—H15B	0.9600
N2—C8	1.342 (9)	C15—H15C	0.9600
			
O14—Cu—N1	85.8 (2)	Mo3—O12—Mo1	92.51 (14)
O14—Cu—N2	174.4 (2)	Mo3—O12—Mo2^i^	91.87 (13)
N1—Cu—N2	99.6 (2)	Mo1—O12—Mo2^i^	163.2 (2)
O14—Cu—N3	93.0 (2)	Mo3—O12—Mo3^i^	104.57 (19)
N1—Cu—N3	177.5 (2)	Mo1—O12—Mo3^i^	97.46 (14)
N2—Cu—N3	81.7 (2)	Mo2^i^—O12—Mo3^i^	97.08 (14)
O1—Mo1—O6	104.5 (2)	Mo3—O12—Mo4	164.8 (2)
O1—Mo1—O11	101.9 (2)	Mo1—O12—Mo4	86.10 (14)
O6—Mo1—O11	100.25 (19)	Mo2^i^—O12—Mo4	85.44 (13)
O1—Mo1—O10	96.6 (2)	Mo3^i^—O12—Mo4	90.65 (12)
O6—Mo1—O10	100.2 (2)	C12—O14—Cu	114.8 (5)
O11—Mo1—O10	147.87 (18)	C11—N1—Cu	110.1 (4)
O1—Mo1—O12	94.64 (17)	C11—N1—H1A	109.6
O6—Mo1—O12	160.6 (2)	Cu—N1—H1A	109.6
O11—Mo1—O12	78.82 (16)	C11—N1—H1B	109.6
O10—Mo1—O12	73.67 (15)	Cu—N1—H1B	109.6
O1—Mo1—O7^i^	164.75 (17)	H1A—N1—H1B	108.2
O6—Mo1—O7^i^	87.88 (19)	C8—N2—C10	119.9 (6)
O11—Mo1—O7^i^	84.26 (18)	C8—N2—Cu	125.9 (4)
O10—Mo1—O7^i^	72.04 (16)	C10—N2—Cu	113.9 (5)
O12—Mo1—O7^i^	72.68 (15)	C1—N3—C9	119.0 (6)
O1—Mo1—Mo3	85.12 (15)	C1—N3—Cu	126.7 (4)
O6—Mo1—Mo3	135.43 (17)	C9—N3—Cu	114.4 (5)
O11—Mo1—Mo3	120.55 (12)	C13—N4—C15	123.2 (7)
O10—Mo1—Mo3	35.24 (11)	C13—N4—C14	119.4 (7)
O12—Mo1—Mo3	41.75 (10)	C15—N4—C14	117.1 (6)
O7^i^—Mo1—Mo3	79.76 (10)	HN1—N5—HN2	92.1
O8—Mo2—O2	105.0 (2)	HN1—N5—HN3	117.9
O8—Mo2—O9	101.5 (2)	HN2—N5—HN3	117.0
O2—Mo2—O9	101.2 (2)	HN1—N5—HN4	125.0
O8—Mo2—O7^i^	100.7 (2)	HN2—N5—HN4	116.8
O2—Mo2—O7^i^	96.4 (2)	HN3—N5—HN4	90.7
O9—Mo2—O7^i^	146.90 (17)	N3—C1—C2	122.9 (7)
O8—Mo2—O10	89.67 (19)	N3—C1—H1	118.5
O2—Mo2—O10	163.20 (16)	C2—C1—H1	118.5
O9—Mo2—O10	83.47 (18)	C1—C2—C3	118.1 (7)
O7^i^—Mo2—O10	72.36 (16)	C1—C2—H2	121.0
O8—Mo2—O12^i^	161.3 (2)	C3—C2—H2	121.0
O2—Mo2—O12^i^	93.45 (17)	C4—C3—C2	119.5 (7)
O9—Mo2—O12^i^	77.44 (15)	C4—C3—H3	120.2
O7^i^—Mo2—O12^i^	73.72 (15)	C2—C3—H3	120.2
O10—Mo2—O12^i^	71.65 (15)	C3—C4—C9	120.4 (7)
O4—Mo3—O5	104.9 (2)	C3—C4—H4	119.8
O4—Mo3—O10	100.12 (19)	C9—C4—H4	119.8
O5—Mo3—O10	96.24 (19)	C6—C5—C10	118.9 (7)
O4—Mo3—O7	102.04 (19)	C6—C5—H5	120.5
O5—Mo3—O7	96.87 (19)	C10—C5—H5	120.5
O10—Mo3—O7	150.32 (16)	C5—C6—C7	121.9 (7)
O4—Mo3—O12	98.9 (2)	C5—C6—H6	119.1
O5—Mo3—O12	156.13 (19)	C7—C6—H6	119.1
O10—Mo3—O12	78.15 (16)	C6—C7—C8	117.0 (7)
O7—Mo3—O12	79.11 (16)	C6—C7—H7	121.5
O4—Mo3—O12^i^	174.24 (19)	C8—C7—H7	121.5
O5—Mo3—O12^i^	80.71 (18)	N2—C8—C7	122.5 (6)
O10—Mo3—O12^i^	77.72 (16)	N2—C8—H8	118.8
O7—Mo3—O12^i^	78.29 (15)	C7—C8—H8	118.8
O12—Mo3—O12^i^	75.43 (19)	N3—C9—C4	120.1 (7)
O4—Mo3—Mo1	89.45 (15)	N3—C9—C10	114.3 (6)
O5—Mo3—Mo1	132.20 (13)	C4—C9—C10	125.5 (6)
O10—Mo3—Mo1	35.97 (13)	N2—C10—C5	119.9 (7)
O7—Mo3—Mo1	124.84 (12)	N2—C10—C9	115.5 (6)
O12—Mo3—Mo1	45.74 (10)	C5—C10—C9	124.6 (6)
O12^i^—Mo3—Mo1	85.72 (10)	N1—C11—C12	110.5 (5)
O13—Mo4—O3	105.1 (2)	N1—C11—H11A	109.6
O13—Mo4—O9^i^	103.1 (2)	C12—C11—H11A	109.6
O3—Mo4—O9^i^	97.9 (2)	N1—C11—H11B	109.6
O13—Mo4—O11	104.12 (19)	C12—C11—H11B	109.6
O3—Mo4—O11	97.0 (2)	H11A—C11—H11B	108.1
O9^i^—Mo4—O11	144.2 (2)	O15—C12—O14	122.6 (7)
O13—Mo4—O5^i^	91.8 (2)	O15—C12—C11	119.1 (6)
O3—Mo4—O5^i^	163.15 (19)	O14—C12—C11	118.2 (6)
O9^i^—Mo4—O5^i^	77.85 (18)	O16—C13—N4	126.8 (8)
O11—Mo4—O5^i^	78.55 (18)	O16—C13—H14	116.6
O13—Mo4—O12	161.5 (2)	N4—C13—H14	116.6
O3—Mo4—O12	93.44 (18)	N4—C14—H14A	109.5
O9^i^—Mo4—O12	73.45 (16)	N4—C14—H14B	109.5
O11—Mo4—O12	73.35 (15)	H14A—C14—H14B	109.5
O5^i^—Mo4—O12	69.71 (15)	N4—C14—H14C	109.5
Mo3—O5—Mo4^i^	118.9 (2)	H14A—C14—H14C	109.5
Mo3—O7—Mo2^i^	109.0 (2)	H14B—C14—H14C	109.5
Mo3—O7—Mo1^i^	108.78 (19)	N4—C15—H15A	109.5
Mo2^i^—O7—Mo1^i^	103.33 (16)	N4—C15—H15B	109.5
Mo2—O9—Mo4^i^	118.6 (2)	H15A—C15—H15B	109.5
Mo3—O10—Mo1	108.8 (2)	N4—C15—H15C	109.5
Mo3—O10—Mo2	110.6 (2)	H15A—C15—H15C	109.5
Mo1—O10—Mo2	104.01 (16)	H15B—C15—H15C	109.5
Mo1—O11—Mo4	117.12 (19)		
			
O1—Mo1—Mo3—O4	−0.51 (19)	O5—Mo3—O12—Mo2^i^	99.5 (4)
O6—Mo1—Mo3—O4	105.2 (3)	O10—Mo3—O12—Mo2^i^	178.02 (19)
O11—Mo1—Mo3—O4	−101.5 (2)	O7—Mo3—O12—Mo2^i^	17.22 (17)
O10—Mo1—Mo3—O4	108.2 (3)	O12^i^—Mo3—O12—Mo2^i^	97.81 (17)
O12—Mo1—Mo3—O4	−103.1 (2)	Mo1—Mo3—O12—Mo2^i^	−163.8 (2)
O7^i^—Mo1—Mo3—O4	−178.49 (17)	O4—Mo3—O12—Mo3^i^	178.77 (16)
O1—Mo1—Mo3—O5	−110.3 (3)	O5—Mo3—O12—Mo3^i^	1.7 (5)
O6—Mo1—Mo3—O5	−4.6 (3)	O10—Mo3—O12—Mo3^i^	80.22 (17)
O11—Mo1—Mo3—O5	148.8 (3)	O7—Mo3—O12—Mo3^i^	−80.58 (18)
O10—Mo1—Mo3—O5	−1.6 (3)	O12^i^—Mo3—O12—Mo3^i^	0.0
O12—Mo1—Mo3—O5	147.1 (3)	Mo1—Mo3—O12—Mo3^i^	98.37 (17)
O7^i^—Mo1—Mo3—O5	71.7 (2)	O4—Mo3—O12—Mo4	−4.0 (7)
O1—Mo1—Mo3—O10	−108.7 (3)	O5—Mo3—O12—Mo4	179.0 (5)
O6—Mo1—Mo3—O10	−3.0 (3)	O10—Mo3—O12—Mo4	−102.5 (7)
O11—Mo1—Mo3—O10	150.3 (3)	O7—Mo3—O12—Mo4	96.7 (7)
O12—Mo1—Mo3—O10	148.7 (3)	O12^i^—Mo3—O12—Mo4	177.3 (7)
O7^i^—Mo1—Mo3—O10	73.3 (2)	Mo1—Mo3—O12—Mo4	−84.4 (7)
O1—Mo1—Mo3—O7	103.8 (2)	O1—Mo1—O12—Mo3	−77.3 (2)
O6—Mo1—Mo3—O7	−150.5 (3)	O6—Mo1—O12—Mo3	92.3 (5)
O11—Mo1—Mo3—O7	2.9 (2)	O11—Mo1—O12—Mo3	−178.6 (2)
O10—Mo1—Mo3—O7	−147.5 (3)	O10—Mo1—O12—Mo3	18.19 (16)
O12—Mo1—Mo3—O7	1.2 (2)	O7^i^—Mo1—O12—Mo3	94.00 (18)
O7^i^—Mo1—Mo3—O7	−74.2 (2)	O1—Mo1—O12—Mo2^i^	27.7 (6)
O1—Mo1—Mo3—O12	102.6 (2)	O6—Mo1—O12—Mo2^i^	−162.7 (6)
O6—Mo1—Mo3—O12	−151.7 (3)	O11—Mo1—O12—Mo2^i^	−73.6 (6)
O11—Mo1—Mo3—O12	1.6 (2)	O10—Mo1—O12—Mo2^i^	123.2 (6)
O10—Mo1—Mo3—O12	−148.7 (3)	O7^i^—Mo1—O12—Mo2^i^	−161.0 (6)
O7^i^—Mo1—Mo3—O12	−75.4 (2)	Mo3—Mo1—O12—Mo2^i^	105.0 (6)
O1—Mo1—Mo3—O12^i^	176.35 (17)	O1—Mo1—O12—Mo3^i^	177.61 (19)
O6—Mo1—Mo3—O12^i^	−77.9 (2)	O6—Mo1—O12—Mo3^i^	−12.8 (6)
O11—Mo1—Mo3—O12^i^	75.39 (19)	O11—Mo1—O12—Mo3^i^	76.36 (18)
O10—Mo1—Mo3—O12^i^	−74.9 (2)	O10—Mo1—O12—Mo3^i^	−86.86 (18)
O12—Mo1—Mo3—O12^i^	73.8 (2)	O7^i^—Mo1—O12—Mo3^i^	−11.05 (14)
O7^i^—Mo1—Mo3—O12^i^	−1.63 (13)	Mo3—Mo1—O12—Mo3^i^	−105.0 (2)
O4—Mo3—O5—Mo4^i^	−178.66 (19)	O1—Mo1—O12—Mo4	87.46 (19)
O10—Mo3—O5—Mo4^i^	−76.4 (2)	O6—Mo1—O12—Mo4	−102.9 (6)
O7—Mo3—O5—Mo4^i^	76.9 (2)	O11—Mo1—O12—Mo4	−13.79 (16)
O12—Mo3—O5—Mo4^i^	−1.7 (5)	O10—Mo1—O12—Mo4	−177.01 (17)
O12^i^—Mo3—O5—Mo4^i^	0.01 (18)	O7^i^—Mo1—O12—Mo4	−101.20 (14)
Mo1—Mo3—O5—Mo4^i^	−75.5 (3)	Mo3—Mo1—O12—Mo4	164.8 (2)
O4—Mo3—O7—Mo2^i^	75.4 (2)	O13—Mo4—O12—Mo3	−176.3 (6)
O5—Mo3—O7—Mo2^i^	−177.7 (2)	O3—Mo4—O12—Mo3	2.7 (7)
O10—Mo3—O7—Mo2^i^	−62.1 (5)	O9^i^—Mo4—O12—Mo3	−94.5 (7)
O12—Mo3—O7—Mo2^i^	−21.57 (19)	O11—Mo4—O12—Mo3	99.0 (7)
O12^i^—Mo3—O7—Mo2^i^	−98.76 (19)	O5^i^—Mo4—O12—Mo3	−177.4 (7)
Mo1—Mo3—O7—Mo2^i^	−22.5 (3)	O13—Mo4—O12—Mo1	98.5 (5)
O4—Mo3—O7—Mo1^i^	−172.6 (2)	O3—Mo4—O12—Mo1	−82.48 (18)
O5—Mo3—O7—Mo1^i^	−65.7 (2)	O9^i^—Mo4—O12—Mo1	−179.72 (18)
O10—Mo3—O7—Mo1^i^	49.9 (5)	O11—Mo4—O12—Mo1	13.75 (16)
O12—Mo3—O7—Mo1^i^	90.45 (19)	O5^i^—Mo4—O12—Mo1	97.42 (15)
O12^i^—Mo3—O7—Mo1^i^	13.26 (16)	O13—Mo4—O12—Mo2^i^	−96.0 (5)
Mo1—Mo3—O7—Mo1^i^	89.5 (2)	O3—Mo4—O12—Mo2^i^	83.04 (17)
O8—Mo2—O9—Mo4^i^	−178.0 (3)	O9^i^—Mo4—O12—Mo2^i^	−14.19 (16)
O2—Mo2—O9—Mo4^i^	−70.0 (3)	O11—Mo4—O12—Mo2^i^	179.28 (19)
O7^i^—Mo2—O9—Mo4^i^	50.9 (5)	O5^i^—Mo4—O12—Mo2^i^	−97.06 (15)
O10—Mo2—O9—Mo4^i^	93.6 (3)	O13—Mo4—O12—Mo3^i^	1.1 (6)
O12^i^—Mo2—O9—Mo4^i^	21.0 (3)	O3—Mo4—O12—Mo3^i^	−179.91 (16)
O4—Mo3—O10—Mo1	−74.8 (2)	O9^i^—Mo4—O12—Mo3^i^	82.86 (17)
O5—Mo3—O10—Mo1	178.8 (2)	O11—Mo4—O12—Mo3^i^	−83.68 (17)
O7—Mo3—O10—Mo1	63.0 (5)	O5^i^—Mo4—O12—Mo3^i^	−0.01 (12)
O12—Mo3—O10—Mo1	22.3 (2)	N1—Cu—O14—C12	2.2 (4)
O12^i^—Mo3—O10—Mo1	99.8 (2)	N3—Cu—O14—C12	−175.7 (4)
O4—Mo3—O10—Mo2	171.6 (2)	O14—Cu—N1—C11	−5.8 (4)
O5—Mo3—O10—Mo2	65.2 (2)	N2—Cu—N1—C11	175.4 (4)
O7—Mo3—O10—Mo2	−50.6 (5)	N1—Cu—N2—C8	4.3 (5)
O12—Mo3—O10—Mo2	−91.3 (2)	N3—Cu—N2—C8	−177.9 (5)
O12^i^—Mo3—O10—Mo2	−13.88 (16)	N1—Cu—N2—C10	178.2 (4)
Mo1—Mo3—O10—Mo2	−113.7 (2)	N3—Cu—N2—C10	−3.9 (4)
O1—Mo1—O10—Mo3	71.8 (2)	O14—Cu—N3—C1	2.1 (5)
O6—Mo1—O10—Mo3	177.9 (2)	N2—Cu—N3—C1	−179.3 (5)
O11—Mo1—O10—Mo3	−53.3 (4)	O14—Cu—N3—C9	−176.9 (4)
O12—Mo1—O10—Mo3	−21.12 (18)	N2—Cu—N3—C9	1.7 (4)
O7^i^—Mo1—O10—Mo3	−97.8 (2)	C9—N3—C1—C2	−0.6 (9)
O1—Mo1—O10—Mo2	−170.27 (17)	Cu—N3—C1—C2	−179.6 (4)
O6—Mo1—O10—Mo2	−64.2 (2)	N3—C1—C2—C3	−0.4 (10)
O11—Mo1—O10—Mo2	64.6 (4)	C1—C2—C3—C4	2.0 (10)
O12—Mo1—O10—Mo2	96.81 (17)	C2—C3—C4—C9	−2.6 (10)
O7^i^—Mo1—O10—Mo2	20.18 (13)	C10—C5—C6—C7	0.5 (11)
Mo3—Mo1—O10—Mo2	117.9 (3)	C5—C6—C7—C8	0.7 (10)
O8—Mo2—O10—Mo3	−166.0 (2)	C10—N2—C8—C7	0.5 (9)
O2—Mo2—O10—Mo3	43.0 (7)	Cu—N2—C8—C7	174.1 (4)
O9—Mo2—O10—Mo3	−64.4 (2)	C6—C7—C8—N2	−1.2 (9)
O7^i^—Mo2—O10—Mo3	92.7 (2)	C1—N3—C9—C4	0.0 (8)
O12^i^—Mo2—O10—Mo3	14.51 (17)	Cu—N3—C9—C4	179.1 (4)
O8—Mo2—O10—Mo1	77.4 (2)	C1—N3—C9—C10	−178.5 (5)
O2—Mo2—O10—Mo1	−73.7 (7)	Cu—N3—C9—C10	0.6 (6)
O9—Mo2—O10—Mo1	178.95 (18)	C3—C4—C9—N3	1.6 (9)
O7^i^—Mo2—O10—Mo1	−23.95 (16)	C3—C4—C9—C10	179.9 (6)
O12^i^—Mo2—O10—Mo1	−102.15 (17)	C8—N2—C10—C5	0.8 (8)
O1—Mo1—O11—Mo4	−72.4 (3)	Cu—N2—C10—C5	−173.6 (4)
O6—Mo1—O11—Mo4	−179.8 (3)	C8—N2—C10—C9	179.7 (5)
O10—Mo1—O11—Mo4	51.4 (5)	Cu—N2—C10—C9	5.4 (6)
O12—Mo1—O11—Mo4	20.0 (2)	C6—C5—C10—N2	−1.3 (9)
O7^i^—Mo1—O11—Mo4	93.4 (3)	C6—C5—C10—C9	179.9 (6)
Mo3—Mo1—O11—Mo4	18.9 (3)	N3—C9—C10—N2	−4.0 (7)
O13—Mo4—O11—Mo1	−179.9 (3)	C4—C9—C10—N2	177.7 (5)
O3—Mo4—O11—Mo1	72.6 (3)	N3—C9—C10—C5	174.9 (5)
O9^i^—Mo4—O11—Mo1	−41.4 (4)	C4—C9—C10—C5	−3.4 (10)
O5^i^—Mo4—O11—Mo1	−90.9 (3)	Cu—N1—C11—C12	8.0 (6)
O12—Mo4—O11—Mo1	−18.9 (2)	Cu—O14—C12—O15	−179.6 (4)
O4—Mo3—O12—Mo1	80.40 (18)	Cu—O14—C12—C11	2.2 (7)
O5—Mo3—O12—Mo1	−96.6 (4)	N1—C11—C12—O15	174.8 (5)
O10—Mo3—O12—Mo1	−18.16 (17)	N1—C11—C12—O14	−6.9 (8)
O7—Mo3—O12—Mo1	−178.96 (19)	C15—N4—C13—O16	178.7 (7)
O12^i^—Mo3—O12—Mo1	−98.37 (16)	C14—N4—C13—O16	5.2 (10)
O4—Mo3—O12—Mo2^i^	−83.42 (18)		

Symmetry codes: (i) −*x*+1, −*y*+1, −*z*+1.

### Hydrogen-bond geometry (Å, °)

**Table:**

*D*—H···*A*	*D*—H	H···*A*	*D*···*A*	*D*—H···*A*
N1—H1A···O3^ii^	0.90	2.32	3.093 (7)	144
N1—H1B···O6	0.90	2.01	2.863 (6)	158
N5—HN1···O1	0.90	2.10	2.888 (7)	146
N5—HN2···O4	0.95	2.13	3.028 (8)	158
N5—HN3···O15^iii^	0.95	1.94	2.761 (8)	143
N5—HN4···O14^iii^	0.98	2.26	3.132 (7)	148

Symmetry codes: (ii) −*x*+1, −*y*+2, −*z*+1; (iii) *x*, *y*, *z*−1.
